# Single Nucleotide Polymorphisms of Leptin and Calpain/Calpastatin in Key Traits of Pork Meat Quality

**DOI:** 10.3390/ani15152270

**Published:** 2025-08-04

**Authors:** Ofelia Limón-Morales, Herlinda Bonilla-Jaime, Marcela Arteaga-Silva, Patricia Roldán-Santiago, Luis Alberto de la Cruz-Cruz, Héctor Orozco-Gregorio, Marco Cerbón, José Luis Cortes-Altamirano

**Affiliations:** 1 Departamento de Biología de la Reproducción, Universidad Autónoma Metropolitana, Unidad Iztapalapa, Mexico City 09340, Mexico; bjh@xanum.uam.mx (H.B.-J.); asm@xanum.uam.mx (M.A.-S.); 2 Departamento de Reproducción, Facultad de Medicina Veterinaria y Zootecnia, Universidad Nacional Autónoma de México, Avenida Universidad, Mexico City 04510, Mexico; patriciaroldan@fmvz.unam.mx; 3 Producción Agrícola y Animal, Universidad Autónoma Metropolitana, Calzada del Hueso 1100, Coapa, Villa Quietud, Coyoacán, Mexico City 04960, Mexico; ladelacruzcc@gmail.com; 4 Ingeniería en Producción Animal, Universidad Politécnica de Francisco I. Madero, Domicilio Conocido s/n, Tepatepec, Francisco I., Madero 42660, Mexico; gohector72@yahoo.com.mx; 5 Facultad de Química, Universidad Nacional Autónoma de México, Mexico City 04510, Mexico; mcerbon85@yahoo.com.mx; 6 Division of Basic Neurosciences, Instituto Nacional de Rehabilitación, “Luis Guillermo Ibarra Ibarra”, Mexico City 14389, Mexico; drjlcortesaltamirano@gmail.com; 7 Departmento de Quiropractica, Universidad Estatal del Valle de Ecatepec, Ecatepec de Morelos 55210, Mexico

**Keywords:** meat quality, calpain/calpastatin, leptin, tenderness, marbling, single nucleotide polymorphisms

## Abstract

The increasing demand for food to meet the needs of the planet’s growing population requires, among other factors, greater and improved meat production. Meat quality is determined by key consumer-preferred traits, particularly tenderness, juiciness, and flavor. Recently, interest has grown in analyzing the genes associated with these phenotypic characteristics. Single-nucleotide polymorphisms (SNPs) are common genomic variations in cattle and represent the most widely used molecular markers. Research on SNP variation is now a major focus of genomic studies aimed at improving meat quality. Leptin levels reflect the amount of adipose tissue in meat, also known as marbling. Several SNPs in the leptin gene and its receptor have been linked to this meat quality trait. Similarly, SNPs in the calpain/calpastatin system play a significant role in postmortem muscle proteolysis and pork tenderness. This review examines these genetic variants as markers involved in the expression of phenotypic traits in meat products and explores their mechanisms of action. Additionally, it provides insights into the genetic variants associated with production-related characteristics.

## Simple Summary

The growing global population demands increased and higher-quality meat production. Meat quality is determined by key traits such as tenderness, juiciness, and flavor, which are influenced by genetic factors. Single-nucleotide polymorphisms (SNPs)—small variations in DNA—are widely studied as genetic markers linked to these traits. For instance, SNPs in the leptin gene and its receptor affect fat distribution in meat (marbling), while variations in the calpain/calpastatin system influence postmortem muscle breakdown and tenderness in pork. This review examines how these genetic markers impact meat quality and explores their biological mechanisms. By understanding SNPs, researchers can identify animals with desirable traits for selective breeding programs.

## 1. Introduction

The global population continues to increase, with the United Nations projecting that the world’s population will reach 9.7 billion by 2050 [1]. This growth entails an ever-greater demand for food, including meat and meat products. Pork is one of the most consumed meats worldwide, accounting for 42% of global red meat production and consumption. Over the past decade, global pork production has grown at an average annual rate of 1.5% [2,3]. In 2023, 78.4% of global pork production was concentrated in three major regions, namely China, the European Union, and the United States, while Mexico ranked eighth, contributing 1.3% of total production [2]. The swine industry plays a significant role in Mexico’s food sector, as pork represents 18.4% of the country’s total meat production and is the third-most consumed meat due to its affordability and quality [4].

Currently, there are approximately 730 pig breeds or lines worldwide, including exogenous and indigenous varieties, with two-thirds found in China and Europe [5]. These breeds vary in size, ranging from large breeds weighing up to 350 pounds to miniature lines. In modern pork production, certain exogenous breeds dominate, including Duroc, Yorkshire (Large White), Landrace, Hampshire, and Pietrain [6]. In Mexico, the most prominent breeds for pork production are crossbreeds of Duroc, Landrace, Hampshire, Chester White, Yorkshire, and Pietrain [4].

To meet the growing demand while also improving pork quality and quantity, programs designed to select for important traits are required. The traditional approach to swine production involves generating commercial hybrids by crossing males that show high meat aptitude with females that have good maternal aptitude, though the goals of selection processes may differ significantly depending on the line or lines involved. While the maternal lines are selected with greater emphasis on features related to maternal aptitude and prolificacy, for the paternal lines, body formation and carcass and meat quality are the most important traits [7]. Conventional breeding programs aimed at improving carcass and pork quality have some disadvantages. For example, the costs and difficulty of phenotypic recording [8], selection decisions based on phenotypical data are often delayed because this process may require that the animals reach slaughtering age; that is, 14 to 20 months.

The emergence of molecular genetics has opened intriguing perspectives regarding the use of genomic information for genetic evaluation in animals. Currently, thanks to our greater knowledge of swine genetics [9]. The study of single-nucleotide polymorphisms (SNPs) relevant to meat production is carried out primarily by traditional and high-throughput methods, which include amplification-refractory mutation system (ARMS), single-step genome-wide association (GWAS), using bi-directional polymerase chain reaction amplification of specific alleles (Bi-PASA) and PCR-restriction fragment length polymorphisms (PCR-RFLP), denaturing strand gradient gel electrophoresis (DGGE), single-stranded conformation polymorphism (SSCP), allele-specific hybridization, allele-specific single-base primer extension, high-throughput assay chemistry, DNA arrays, and pyrosequencing [10].

Various molecules participate in regulating the productive characteristics of pork, including the amount of fat and tenderness, two traits of special interest to consumers. This review focuses on leptin and the calpain/calpastatin system and their relationship with traits associated with meat quality. The objective is to analyze existing SNPs of leptin and the calpain/calpastatin system related to important productive traits and quality in pork.

## 2. Materials and Methods

**Figure 1:**
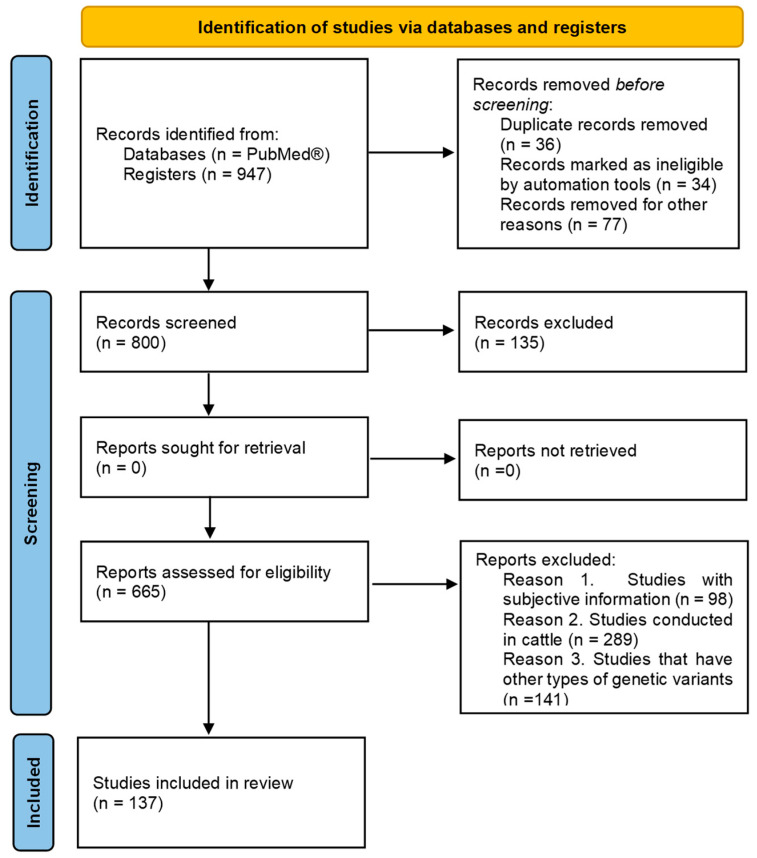
The search protocol, including included or excluded sources. Adapted from Page et al. [11].

This systematic review was written following the Preferred Reporting Items for Systematic Reviews and Meta-Analyses (PRISMA) [11] (Figure 1).

### 2.1. Exclusion Criteria

Studies over a period from 2000 to 2024 were prioritized; works conducted in pigs and studies that have other types of genetic variants. Duplicate records were eliminated. Studies were considered that showed statistical analysis linking the SNP with meat quality characteristics, with a significance of at least *p* < 0.05.

### 2.2. Information Sources, Search, and Selection

We analyzed 137 peer-reviewed publications in English in PubMed^®^; due to the limited research on this topic on meat quality in pigs, only 37 works were considered. These studies were conducted on SNPs of LEP/LEPR or CAPN1/CAST, dated between 2002 and 2024. The search terms were (pork* OR pig* OR pork quality* OR meat quality) AND (leptin* OR leptin receptor*) AND (marbling* OR intramuscular fat* OR fatty acid*) AND (calpain* OR calpastatin* OR CAPN1* OR CAST*) AND (Tenderness* OR postmortem proteolysis*) AND (SNP*). In addition, the use of * in terms allows for broadening the search results. The search terms were used in PubMed^®^: title, abstract, and keyword (TITLE-ABS-KEY) parts of documents.

## 3. Results

### 3.1. Importance of SNPs to Meat Quality

The presence of genetic markers in genes associated with economically important traits—including production, reproduction, and meat quality characteristics—enables researchers to identify quantitative trait loci (QTL). QTL analysis facilitates the genotyping of individual animals, allowing for the detection of genetic variants linked to desirable traits and the subsequent development of more efficient breeding programs [12].

In swine, numerous QTLs have been identified for various economically relevant traits such as growth rate, marbling, carcass quality, meat quality, and prolificacy. For instance, total fat content and backfat thickness are key parameters significantly influencing carcass quality. Multiple QTLs have been associated with these traits, along with potential candidate genes involved in adiposity regulation. Notably, several well-characterized genes related to energy metabolism, such as the leptin gene, have been identified as promising markers for breeding programs [13].

Single-nucleotide polymorphisms (SNPs) represent the most prevalent form of genetic variation and have become the molecular marker of choice in genomic studies of swine and cattle. These variations involve single-nucleotide substitutions, resulting in simple biallelic polymorphisms [14].

In pigs, traits related to meat quality, such as color, tenderness, marbling, and juiciness, greatly influence consumer purchasing decisions, so research designed to improve meat quality in these areas has intensified. Today, many genes associated with certain characteristics of meat quality have been identified [15], and recent studies have established a relationship between candidate gene SNPs and key features of carcasses and meat quality [16,17,18]. These include leptin and calpastatin.

### 3.2. Leptin as a Marker of Marbling and Meat Quality in Pork

Intramuscular fat (IMF) and backfat thickness are important traits in pigs, influencing meat quality and economic value. IMF, the fat within the muscle affects pork flavor, tenderness, and juiciness. Backfat thickness, the subcutaneous fat layer, is linked to carcass leanness and economic value. Selecting leaner pigs (thinner backfat) can sometimes reduce IMF, potentially impacting meat quality [19,20,21]. Backfat and IMF, often referred to as marbling, are distinct fat deposits in animals, but they are related. Backfat is the subcutaneous fat layer, while IMF is the fat deposited within the muscle tissue. Marbling is a measure of balance among the synthesis, breakdown, and absorption of triglycerides. In pork, sensory characteristics, tenderness, and water retention are all closely related to the degree of marbling [22]. The juicy flavor and texture of this meat are generally associated with a percentage of marbling/IMF ˃2.5% [23,24]. Triglycerides are stored primarily in adipocytes but are also found between muscle fibers in the form of droplets [25].

Numerous studies have demonstrated that genes regulating fat metabolism significantly influence pork intramuscular fat content. Enhanced expression of genes involved in fatty acid synthesis and lipid metabolism correlates with elevated intramuscular fat deposition across various pig breeds. Genes that are related to lipid metabolism and fatty acid synthesis in pigs include adiponectin (*ADIPOQ*), adiponectin receptor 1 (*ADIPOR1*), acetyl-CoA carboxylase alpha (*ACACA*), acyl-CoA oxidase 1 (*ACOX1*), catalase (*CAT*), diacylglycerol acyltransferase 1 and 2 (*DGAT1* and *DGAT2*), fatty acid binding, muscle and heart (*FABP3* and *H-FABP*), fatty acid binding protein 4, adipocyte (*FABP4* and *A-FABP*), fatty acid synthase (*FASN*), hormone-sensitive lipase (*LIPE* and *HSL*), lipoprotein lipase (*LPL*), peroxisome proliferator-activated receptor alpha and gamma (*PPARA* and *PPARG*), retinoid X receptor gamma (*RXRG*), solute carrier family 2 member 4 (facilitated glucose transporter) (*SLC2A4* and *GLUT4*), and sterol regulatory element-binding transcription factor 1 (*SREBF1* and *SREBP-1C*), leptin and leptin (*LEP*) receptor (*LEPR*) among others [26]. Due to its importance, we will focus on leptin and its receptors.

Leptin is a 16 kDa peptide hormone secreted mainly by white adipose tissue. It circulates in serum as both a free and protein-bound entity [27,28]. In mammals, leptin participates in regulating key corporal functions, such as energy balance, homeostasis, body weight, and the mobilization of fat. For these reasons, leptin deficiency leads to increased body fat mass due to excessive food intake. Leptin is an anorexigenic hormone, meaning it suppresses appetite. Leptin activates anorexigenic hypothalamic neurons and, at the same time, inhibits orexigenic neurons, which promote food intake [28], and its levels reflect the amount of adipose tissue present and nutritional states [29]. The central function of leptin is to inhibit food intake and stimulate thermogenesis to maintain energy homeostasis [30].

In the hypothalamic arcuate nucleus, leptin binds to receptors on two groups of neurons: the first is part of the orexigenic pathway and consists of neurons that release NPY and neurons that produce agouti-related protein (AGRP); the second is part of the anorexigenic pathway and includes neurons that secrete propriomelanocortin (POMC) and α-melanocyte-stimulating hormone (α-MSH). Leptin activates α-MSH/CART neurons and inhibits NPY/AGRP neurons [30].

Obesity is characterized by hyperleptinemia (elevated leptin levels) and leptin resistance, a state of reduced tissue sensitivity to leptin due to impaired signaling pathways [31,32]. One contributing factor to leptin resistance is mutations in the leptin receptor (LEPR) gene. Studies indicate that increased LEPR expression correlates with higher circulating leptin levels and enhanced fat deposition [33,34].

Leptin is one of the biomolecular markers most closely associated with high-performance individuals, as it fosters greater adaptability and productivity [35] through its effects on appetite, growth, energy, metabolism, reproduction, and disease resistance [36]; thus, some studies have proposed that leptin levels could be an marker for selection programs designed to improve feed efficiency, growth, body fat deposits and meat and carcass quality in swine [37,38,39].

#### 3.2.1. Structure

Leptin is produced by the *LEP* gene—or the *Ob* gene—which is located on chromosome 18 in pig ATP/ADP s. This gene codes for a peptide made up of 167 amino acids that weighs approximately 16kD [40]. The sequence of the *LEP* gene is highly conserved in mammals, showing about 65% similarity among such varied species as humans, gorillas, chimpanzees, cattle, pigs, rats, and mice [41,42]. Leptin’s structure includes four antiparallel α-helices (called A, B, C, D). AB and CD are connected by two long cross-links, and a short loop connects BC. Together, they form a left-handed helical bundle. The presence of exposed hydrophobic residues in its structure is crucial for receptor binding. In addition, these hydrophobic areas increase the molecule’s tendency to self-associate and form aggregates [43].

The LepR has six different isoforms produced through alternative splicing of the LepR gene (or the Db gene) located on chromosome 6 [33]. These isoforms are called LepRa, b, c, d, e, and f. They function as transmembrane receptors with a box 1 motif that is required for the attachment of Janus kinase 2 (JAK2). Although these isoforms share similar extracellular and transmembrane domains, their intracellular domains differ. All six have the same N-terminal extracellular domain that interacts with leptin, composed of 816 amino acids and 4 cysteine residues [44]. They feature an intracellular domain at the carboxyl terminal that can vary from 32 to 40 amino acids or 303, depending on whether it is a long or short isoform [45]. The isoforms are categorized as short (LepRa, c, d, and f), long (LepRb), and secreted (LepRe) [45]. The long form—LepRb—has a complete intracellular domain of approximately 300 amino acids that participates in leptin signaling [27]. The LepRe isoform is soluble and can bind circulating leptin, thus hindering its transport to the central nervous system. It lacks transmembrane and cytoplasmic domains [45]. The long isoform can be phosphorylated in its intracellular domain at three distinct tyrosine sites by activating JAK2. It plays a key role in leptin’s effects on energy regulation and neuroendocrine functions [40,45].

#### 3.2.2. Action Mechanism

The leptin-LEPR signaling axis influences bone marrow mesenchymal stromal cell differentiation, promoting adipogenesis over osteogenesis through hypothalamic neuron modulation via the JAK2/STAT3 pathway [46,47]. The long form of the leptin receptor (LEPRb) lacks intrinsic enzymatic activity but is associated with Janus kinase 2 (JAK2), a cytoplasmic tyrosine kinase [27,48,49].

LepR activation may engage various signaling pathways. LepRb is a class I cytokine receptor with no innate kinase activity. Upon binding to leptin, it recruits and activates JAK2, which autophosphorylates on three tyrosine residues on LepRb (Y985, Y1077, and Y1138). Each phosphorylation site is linked to a specific physiological role of leptin, as follows:

– Y985 activates Src homology domain protein-2 (SHP-2) and mitogen-activated protein kinase (MAPK) signaling to provide negative feedback in the leptin signaling pathway.
– Y1077 triggers the signal transducer and activator of transcription-5 (STAT5) signaling to mediate leptin’s reproductive effects.
– Y1138 activates STAT3 signaling, which plays a central role in leptin’s effects on energy balance and neuroendocrine functions [45]. It is well known that other pathways can be activated, depending on the type of tissue and its specific function.

#### 3.2.3. Leptin in Marbling

As mentioned above, the fat content of meat is an important characteristic for consumers since, for example, the polyunsaturated fatty acids (PUFAs) in adipose tissue reduce the risk of certain diseases and are beneficial for cardiovascular health [50]. The deposition of IMF in animals occurs mainly from the fetal stage to weaning [51,52], while accumulation in adults is a process that involves the proliferation and differentiation of preadipocytes [53].

Recent studies have shown that leptin inhibits the proliferation of intramuscular preadipocytes [52], so it is known to stimulate fatty acid oxidation and esterification in muscle, both in vitro and in rodents [54]. This increases the body’s capacity to hydrolyze intramuscular triacylglycerol but decreases its ability to transport FA across the sarcolemma [55]. Leptin also reduces triacylglycerol reserves in skeletal muscle [55]. Consistent with this, exogenous leptin administration in rats has been shown to diminish fat deposition by directly inhibiting the proliferation and/or differentiation of preadipocytes [56] through various signaling pathways: ERK1/2, MAP kinase calcium calmodulin-dependent protein kinase, (CaMKK2)/5′-AMP-activated protein kinase, the (AMPK)/acetyle-CoA carboxylase (ACC) pathway, and AK/STAT [57,58]. The AMPK pathway is the main one involved in leptin’s effects on fatty acid metabolism [57,59]. It also promotes adipocyte reconstruction [60]. Leptin activates AMPK by stimulating FA oxidation through two distinct mechanisms: early and delayed effects. The former are independent of sympathetic activity with direct activation of AMPKα, while the latter involve hypothalamic neurons that activate AMPKα in the target organ [57].

A lower ATP/ADP ratio acts as an intracellular sensor of energy levels, which activates the AMPK pathway [61], a serine/threonine protein kinase that is activated in response to stressors that reduce intracellular ATP levels (e.g., fasting, hypoglycemia, and exercise [61]). When activated in skeletal muscle, it stimulates FA oxidation and glucose uptake [62]. AMPK is a heterotrimer that presents one catalytic subunit (α) and two regulatory subunits (β and γ). Leptin is known to specifically activate AMPK α2 by AMP, through phosphorylation of a threonine residue in the α catalytic subunit [57]. In this case, Lep promotes an increase in the phosphorylation level of AMPKα by increasing the protein expression of CAMKK2 [52].

Other studies performed by GWAS have demonstrated that leptin inhibits triglyceride accumulation and fat droplet formation because it downregulates the expression of SREBF1 by activating the AMPK signaling pathway, resulting in the downregulation of FASN and ACCα expression [52]. SREBF1 is a transcription factor that promotes the expression of adipogenic genes such as *ACCα* and *FASN* [60], which may increase triglyceride accumulation and lipid droplet formation [63].

#### 3.2.4. SNPs in Leptin (LEP) and the Leptin Receptor (LEPR) in Relation to Meat Quality

Various SNPs in the *LEP* gene and its receptor have been discovered in numerous pig breeds and hybrids through different techniques such as SSCP, BiPASA, PCR-RFLP, and GWAS. Some of these have been assessed for their impact on productive traits and meat quality (Table 1). The present study focuses solely on the SNPs that influence the latter factor. In most of the SNPs evaluated to date, and in most of the breeds examined, one of the alleles at each location exhibited a low-to-very low occurrence. SNPs g.867 C>T have been linked to average daily gain and feed efficiency and backfat thickness in Duroc pigs (*p* < 0.05), but not in Landrace or Yorkshire breeds [64]. Meanwhile, in other studies with PCR-RFLP, the SNPs g.2845 A>T and g.3996 T>C have been correlated with total feed consumption during growth and weight gain in Landrace pigs (*p* < 0.0078), though these associations were not observed in Duroc and Yorkshire animals [65]. Different studies show that the SNPs g.3469 T>C have been statistically significant related to such key production parameters as abdominal fat, backfat thickness, IMF, loin weight, meat content, ham weight, and ham cut weight in Duroc, Hampshire, Landrace, and Large White pigs [64,65,66,67,68,69].

SNP c. 2863 G>A is ubicated in the distal promoter region of the leptin gene and was genotyped by the PCR-SSCP approach in pigs of the Duroc, Yorkshire, Laiwu, Lulai Black, and Landrace/Yorkshire crossbreds. That study showed a relationship between higher levels of leptin and its mRNA in the serum and backfat of the animals with the GG genotype than in those with the GA or AA genotypes, but only in Landrace/Yorkshire crossbreds and Lulai Black pigs. The authors thus proposed these SNPs as a potential DNA marker for backfat [22]. In other research, the SNP rs45431504 (c.289T>C) in *LEP* was measured by pyrosequencing; this SNP was found to be associated with changes in fat deposits in the skeletal muscle (*p* < 0.001) of Polish Large White pigs [70]. In this vein, a study of an experimental Iberian/Landrace crossbreed found that the g.1387C>T and g.1382C>T intron SNPs are linked and have additive effects on both live and carcass weight, as well as dominant effects on backfat and saturated fatty acid content and increased growth (*p* < 0.05), possibly due to greater voluntary feed intake [71]. There is evidence that the SNPs in *LEP* may also be related to the desaturation of fatty acids into monounsaturated fatty acids (MUFAs) [47,72], while non-synonymous mutations in *LEP* may foster changes in FA composition in muscle.

*LEPR* is expressed in the brain and peripheral tissues, including muscle and white adipose tissue [52,73,74]. It has been analyzed for SNPs related to marbling in pork and its quality. Four SNPs were found in intron 2 and exons 2, 6, and 18 in Yorkshire, Landrace, and Duroc pigs. The SNPs in exons 6 and 18 were found to be related to backfat in Landrace and Yorkshire pigs, respectively, whereas the polymorphisms in exon 18 were related to feeding efficiency in Duroc animals [75]. *LEPR* SNPs c.2002C>T (genotyped by pyrosequencing), located in exon 14, showed strong associations with backfat in Iberian × Meishan and Landrace pig populations, and demonstrated that the T allele in c.2002C>T is a marker for a tendency toward obesity [75,76,77].

**Table 1:** SNPs of leptin and leptin receptors in different pig lines and crosses related to productive traits.

Single Nucleotide Polymorfism	Population	Productive Trait	Method	Significance	Ref.
**Leptin**				**Chromosome 18**	
g. 867 C>T	Duroc, Hampshire, Landrace and Large White pigs.	Backfat thicknes	Bi-PASA and PCR-RFLP assays	*p* < 0.001	[65]
Exon 3 C>T	Landrace and Yorkshire	Average daily weight gain and feed efficiency	PCR-RFLP	*p* < 0.05	[44]
Intron 1 C>T	Duroc	Backfat thicknes	PCR-RFLP	*p* < 0.05	[44]
2845 A>T	Landrace	Total feed consumption during growth and weight gain	PCR-RFLP	*p* < 0.0078	[13]
3996 T>C	Landrace	Total feed consumption during growth and weight gain	PCR-RFLP	*p* < 0.0078	[13]
rs324640280 (c.339C>T)	Duroc	Subcutaneous fat	RFLP and SSCP	Without association	[69]
g.3469 T>C	Duroc, Hampshire, Landrace and Large White	Abdominal fat, backfat thickness, intramuscular fat, meat content, loin weight, loin muscle area, ham weight, and ham cut weight	RFLP and SSCP	*p* < 0.0078	[13,69]
c. 2863 G>A	Duroc, Yorkshire, Laiwu, Lulai Black and Landrace/Yorkshire crossbreds	Leptin levels in serum and backfat	PCR-SSCP	*p* < 0.01	[22]
LEP g.1387C>T LEP g.1382C>T	Experimental Iberian/Landrace crossbred	Weight (live and carcass), backfat thickness and saturated fatty acid content in fat, increased growth, increased voluntary feed intake	Pyrosequencing	*p* < 0.05	[71]
rs45431504 (c.289T>C)	Polish Large White	Changes in fat deposits in skeletal muscle	Pyrosequencing	*p* < 0.001	[70]
**LEPR**				**Chromosome 6**	
c.2002C>T	Landrace, Yorkshire and Duroc		MS-PCR and PCR-RFLP	*p* < 0.05	[44]
	Iberian × Meishan and Landrace	Backfat thickness	Pyrosequencing, and PCR-RFLP	*p* < 0.01	[75,76,77]
c.2002C>T	Duroc Duroc	Backfat thickness, fat area ratios, serum leptin concentration Growth rate and fat deposition	PCR–RFLP and SSCP	*p* < 0.01	[34,78]
c.232A>T	Polish Landrace	Backfat thickness	Sequenom MassARRAY	*p* < 0.01	[79]
c.232A>T	Duroc × Landrace Yorkshire × Maternal Landrace dams with Duroc	Serum leptin concentrations	Sequenom MassARRAY	*p* < 0.01	[80]
c.2856C>T	Ukrainian white pigs	Backfat thickness and average daily weight gain	PCR–RFLP	*p* < 0.05	[81]

Recently, a relation was found in a pure Duroc line between this SNP and backfat, fat area ratios, and serum leptin concentrations [33] coupled with an influence on growth rates and fat deposition [78]. These SNPs can also exert an effect with three others on exon 4 (c.221C>T, c.232A>T, c.233T>C) analyzed by RFLP and genotyped using a primer extension assay [33]. Other research groups found a similar relation between the SNP c.232A>T and backfat in Polish Landrace pigs [79]. Finally, these SNPs were linked with changes in serum leptin concentrations in two pig lines when White dams (Yorkshire × Maternal Landrace) were crossed with Duroc or Landrace boars and when the progeny of Duroc- and Landrace-sired lines were crossed [80]. Studies of native breeds, like large Ukrainian white pigs, have found that the LepR SNPs c.2856C>T are associated with both backfat and average daily weight gain [81].

Taken together, these studies show that the SNPs in both leptin and its receptor can be considered genetic markers for the deposition of subcutaneous fat and average daily weight gain, findings that make them potentially useful in selection processes (Table 1).

### 3.3. The Calpain–Calpastatin System as a Marker of Tenderness and Quality in Pork

Another important aspect of meat quality is post-mortem muscle proteolysis due to its impact on such quality traits of pork as color, taste, texture, and tenderness, among others. The latter is considered an essential parameter of the eating quality of meat [82,83]. In this regard, some authors have mentioned that the calpain system and its inhibitor—calpastatin (CAST) [84,85]—are related to post-mortem muscle proteolysis and meat tenderization [86,87]. The tenderness of meat depends on the degree of alteration of the muscle structure and the associated proteins, in addition to the CAST system. It is known that there are other enzymes that participate in the degradation of proteins, such as cathepsins, proteasome, and caspase [88].

Calpains are intracellular calcium-dependent, cysteine proteases that are widely distributed in vertebrate organisms [89]. Their activity is regulated by calcium, and they were identified as activators of phosphorylase b kinase [90] and protein kinase C [91]. In addition, the main role of calpain is to cleave multi-domain enzymes to produce fully active enzymes [92]. The phosphorylation state of calpastatin, influenced by enzymes like protein kinase A (PKA), can affect its ability to inhibit calpain. Phosphorylation can enhance calpastatin’s inhibitory effect, potentially leading to less tender meat. Also, the type of muscle (slow-twitch or fast-twitch) and muscle fiber composition can influence calpain and calpastatin expression and activity, thus affecting meat tenderness [88].

The calpain–calpastatin system in skeletal muscle consists of two enzymes called typical or ubiquitous calpain isoforms: calpain 1 (CAPN1) and calpain 2 (CAPN1). Both are 80 kDa proteins whose activity requires heterodimerization with the calpain small subunit (28 kDa). After heterodimer formation, they are called μ-calpain and m-calpain [93,94]. Calpain 3 is the skeletal muscle-specific isoform. It is not associated with a small subunit but can form homodimers [94]. The most abundant isoforms in skeletal muscle are μ-calpain and calpain 3.

#### 3.3.1. Structure

The two common forms of calpain (µ-calpain, m-calpain) share a similar structure [95,96,97,98]. These forms are heterodimers, each one consisting of a large catalytic subunit (80 kDa) and a smaller regulatory subunit (28 kDa) that undergoes autolysis in the presence of calcium [99]. These proteins are highly conserved in vertebrates, as over 90% of their amino acid sequences are identical [95]. The large subunit is organized into four domains:

1. Domain I, the N-terminal domain, is hydrophobic and participates in triggering proteinase activity.
2. Domain II is the active site. It has similarities with the typical structure of thiol proteinases, including essential amino acid residues like Cys and His/Asn [95,96,97,98].
3. Domain III contains amino acid residues that are crucial for interaction with specific ligands [98]. It is thought to bind to phospholipids, potentially interacting with cell membranes [100].
4. Domain IV has four calcium-binding sites like calmodulin [93,98].

The 28 kDa subunit participates in modulating the catalytic activity of the proteinase. It consists of two domains: V and VI. Domain V contains a glycine-rich sequence that is thought to bind to membrane phospholipids. It is referred to as the hydrophobic domain. Domain VI shares a high structural similarity with calmodulin and includes four calcium-binding sites [93,96].

CAST, in contrast, is an intrinsic calpain inhibitor prevalent in vertebrates [92]. Studies have shown approximately a 65% amino acid sequence similarity among calpastatins from distinct species. Unlike calpains, calpastatins have multiple isoforms, even within the same tissue [101]. Though they derive from a single gene located on chromosome 2 in pigs [102], distinct promoters [103,104,105] and alternative splicing [106,107,108] produce various calpastatin isoforms with molecular masses that range from 17.5 [105] to 46.35 kDa [104] and even 84 kDa [103].

Initially identified in mice, calpastatins are classified into four types—I, II, III, IV—based on their exon sequences and NH2-terminal alternative splicing [93].

- Type I begins at exon 1xa, 1y, and 1z and is expressed in the brain, liver, and testes of mice.
- Type II begins at exon 1xb, 1y, and 1z and is primarily expressed in skeletal and cardiac muscle.
- Type III begins at exon 1u and encodes the prototypical calpastatin, which is widely present in mouse tissues, but is also observed in the cardiac and skeletal muscle of pigs [106].
- Type IV begins at exon 14t and is unique, as it is found only in the testes with no expression in other calpastatins.

As mentioned previously, calpain 3 is a 94 kDa polypeptide with sequence homology to the large subunits of the ubiquitous calpains (l- and m-calpain) that does not bind to small subunits [109].

Regarding the amino acid sequence, we found that calpastatin isoforms containing an L and/or XL domain are present in various tissues of several species. The XL domain contains three sites that can be phosphorylated by protein kinase A. The XL and L domains are followed by domains I, II, III, and IV. Within each of the latter, there are three subdomains, called A, B, and C [93]. Some reports suggest that each one of the individual calpastatin domains—I, II, III, and IV—can inhibit one calpain molecule. Domain IV of calpain binds to subdomain A of calpastatin, domain II near the active site of calpain binds to subdomain B—essential for inhibitory activity—while domain VI binds to subdomain C [93].

#### 3.3.2. Action Mechanism

The action mechanism of the calpains derives from their presence in the cytosol in an inactive form. When intracellular free calcium increases, the enzyme is translocated to the membranes. Once bound there, calpain undergoes an autoproteolysis process that generates the active form. During this process, the hydrophobic domain is removed from both calpain subunits, which causes the release of the active calpain into the cytosol by decreasing their affinity for the membrane [98,110]. However, if autoproteolysis continues, the enzyme is inactivated [98].

The action mechanism of the calpastatins was established through crystallographic analysis: calpastatin action mechanism involves inhibiting calpain by occupying both sides of the active site cleft, so this recognition is calcium-dependent. Calpastatin passes through the active site cleft of calpain, which is opened by its binding to calcium. It does so uncleaved by surrounding the active cysteine site [111]. The inhibitory domain of calpastatin recognizes only the calcium-bound form of calpain [111,112].

#### 3.3.3. The Calpain–Calpastatin System and Meat Tenderness

The calpain–calpastatin system performs several functions in cells: regulating gene expression; remodeling cytoskeletal junctions to the plasma membrane during motility and cell fusion; the cleavage of proteins in signal transduction pathways; and degrading enzymes that control progression through the cell cycle, among others [93]. In muscle tissue, specifically, it is linked to such functions as apoptosis, myogenesis, cell signaling, cell differentiation, and protein modification related to their degradation through targeting the proteasome degradation pathway. Finally, μ-calpain in skeletal muscle reduces calcium release from the sarcoplasmic reticulum, following periods of excessive release, and can prevent major degradation [94].

During muscle proteolysis, an increased autoproteolysis of the calpain catalytic subunit occurs [113], reducing it to a 76 kDa form that has a reduced calcium requirement for its activity [93]. Thus, the earlier onset of autolysis is associated with a degradation of muscle protein [114]. In this sense, μ-calpain is the most important isoform in post-mortem proteolysis and meat tenderization [88,113,114,115,116,117].

In relation to skeletal muscle, μ-calpain induces the cleavage of myofibrillar proteins, including such cytoskeletal proteins as troponin-T, tropomyosin, vinculin, dystrophin, α-actinin, titin, fodrin, and desmin, as well as the sarcolemma-associated protein complex spectrin [88,94,117,118]. This suggests that μ-calpain participates significantly in sarcomeric organization and/or postmortem disassembly and, hence, in the tenderness of muscle tissues [94,118].

Several studies have shown that calpain 3 is not the major contributor to meat tenderness, even though it may exist in higher concentrations in skeletal muscle. Studies in knockout mice show that calpain 3 is not involved in postmortem skeletal muscle proteolysis [119]. In contrast, μ-calpain is directly involved in the degradation of important skeletal muscle proteins. Studies of μ-calpain knockout mice have demonstrated this implication for postmortem tenderness [118].

Calpastatin specifically inhibits μ-calpain and m-calpain. It binds and inhibits calpains in a calcium-dependent manner [120,121]. High calpastatin activity is related to decreased proteolysis, increased tenderness, and protein turnover after slaughter [88,93,117]. In this vein, reports on callipyge lambs have observed high levels of calpastatin and shown greatly reduced proteolysis rates and post-mortem meat tenderization [113]. Overexpression of calpastatin in mice reduces postmortem proteolysis of skeletal muscle [117], suggesting an important role of the calpastatins in the degradation of muscle proteins and, thus, in meat tenderness.

#### 3.3.4. SNPs in Calpain (CAPN1) and Calpastatin (CAST) in Relation to Meat Quality

Some of the SNPs have been identified using techniques such as RFLP, microarrays in the calpain sequence are associated with pork tenderness. There is extensive evidence of μ-calpain (*CAPN1*) and calpastatin (*CAST*) SNPs in various breeds of cattle in relation to meat tenderness and other characteristics, such as juiciness and flavor [122,123,124,125]. In the case of swine, however, few studies have explored the association of *CAPN1* with traits of economic interest in meat (Table 2).

A sequence analysis was performed for this gene to search for SNPs. The authors found eight, but only three resulted in nonsense mutations and amino acid changes at amino acid position 54 (serine/threonine), position 192 (glycine/glutamic acid), and valine/isoleucine at position 363, in Yorkshire and Min pigs and wild boars in different proportions. That study, however, did not find an association with any specific characteristics of meat [126]. Other research genotyping using the Illumina Porcine SNP60 BeadChiph identified candidate SNPs from the previous study located in *CAPN1* with a statistically significant association (*p* < 0.05) with meat tenderness and shear strength in a multigenerational Landrace–Duroc–Yorkshire composite population [127].

In contrast, *CAST* has been widely analyzed in relation to SNPs in this gene related to various characteristics of economic interest in pork. Some association studies have implicated *CAST* sequence variations as genetic markers that may influence meat tenderness and pH in hybrid crosses of (Polish Large White × Polish Landrace) × (Hampshire × Pietrain) [128]. In 2004, Ciobanu and cols. identified several missense and silent mutations in *CAST* in the F2 of a Berkshire × Yorkshire family, and *CAST* Ser66Asn and Ser638Arg substitutions in the phosphorylation of *CAST* by PKA. Ser66Asn is found in a conserved area of domain L, while Arg249Lys and Ser638Arg exist in, or near, subdomain C [129,130]. The haplotype was significantly associated (*p* < 0.01) with loss during cooking and higher juiciness, tenderness, and, therefore, better meat quality. This same haplotype was subsequently analyzed in the Duroc–Landrace–Yorkshire swine lines for an association with longissimus shear force, an indicator of tenderness (*p* < 0.0005) at 7- and 14-days postmortem [131]. Subsequently, other research groups found a relation between this haplotype and SNPs in markers such as *ADIPOQ*, *FTO*, *TNF*, *LEPR*, *AMPD1*, *MC4R*, and *DGAT1*, in association with characteristics related to meat quality, including pH, color, and cutting force, and sensory traits like appearance, tenderness, flavor, juiciness, and carcass weight in various breeds and hybrid lines [9,132,133,134].

Studies have associated SNPs with the intron variant *CAST*_rs196949783G>A, mainly in relation to intramuscular fat content, water retention capacity, pH, firmness, toughness, and the weight of the *Longissimus thoracis et lumbar* muscles [8]. Alves et al. (2017) detected the participation of g.5669 T>C and g.49346C>T in loin thickness in Duroc × Iberian crosses and pure Iberian pigs, as well as in the shear force in the loin of crossbred animals [135].

This evidence highlights the importance of this marker for the trait of meat tenderness and its potential usefulness in selection programs (Table 2).

**Table 2:** SNPs of CAPN1 and CAST in different pig lines and crosses related to productive traits.

Single Nucleotide Polymorfism	Population	Productive Trait	Method	Significance	Reference
**CAPN1**				**Chromosome 6**	
Not reported	Yorkshire pig, Min pig and wild boar	Not reported	Not reported	Not reported	[126]
g.157T>C	Italian Duroc × (Landrace × Large White) crossbred	Larger myofibril diameter, meat redness	PCR-RFLP	*p* < 0.0001	[83]
rs81358667G>A	Iberian pigs	Shear force and cooking losses	KASP-PCR	*p* < 0.05	[8]
**CAST**				**Chromosome 2**	
CAST 66 Ser > Arg CAST 249 Arg>249Lys CAST 638Ser>638Arg	Berkshire × Yorkshire crossbreed Duroc–Landrace–Yorkshire swine lines Mexican creole pigs, as well as in the Yorkshire breed	Cooking loss, juiciness and tenderness Shear force (tenderness) Soft and juicy meat	PCR-RFLP and Sequenom MassARRAY	*p* < 0.05–*p* < 0.001	[129,130]
g.76872 G>A	Italian Duroc × (Landrace × Large White) crossbred	Drip loss and pH	PCR-RFLP	*p* < 0.0001	[83]
g.5669 T>C g.49346 C>T	Duroc × Iberian crosses and pure Iberian pigs	Thickness and shear force in muscle	Sanger sequencing	*p* < 0.05–*p*< 0.01	[135]
rs196949783 G>A	Polish Landrace, Polish Large White, Pietrain and Duroc Iberian pigs	Intramuscular fat content, water holding capacity, pH, firmness and toughness and the weight of the Longissimus thoracis et lumbar	KASP-PCR	*p* < 0.05	[8]

## 4. Discussion

Enhancing meat production and quality remains a complex and gradual process, yet the application of animal genetic evaluations offers a promising avenue for optimization. Meat quality determinants can be categorized into two principal factors: genetic (heritable) components and environmental or management-related influences. Heritability estimates serve as crucial indicators for predicting selection response, with intramuscular fat (IMF) content in swine demonstrating moderate-to-high heritability (0.39–0.65) and tenderness showing comparable values (approximately 0.45), underscoring the significant genetic contribution to these economically important traits [136,137]. Contemporary breeding strategies increasingly utilize marker-assisted selection (MAS) to identify and propagate favorable alleles associated with production traits, thereby improving selection efficiency while reducing temporal and financial expenditures compared to conventional methods [26].

Nevertheless, the phenotypic expression of gene polymorphisms exhibits considerable interbreed variability, and inconsistencies persist among studies correlating single-nucleotide polymorphisms (SNPs) with productive trait enhancements. This variability stems from the pleiotropic nature of genes operating within complex metabolic networks, where genetic modifications may yield unintended cascade effects. Furthermore, epigenetic regulation, nutritional inputs, and pre-slaughter environmental stressors constitute additional determinants of ultimate meat quality [137]. While MAS represents a transformative tool for genetic improvement, its implementation necessitates large sample sizes to mitigate spurious associations and functions most effectively as a complement to—rather than a replacement for—traditional selection paradigms in identifying superior genotypes [26]. This implies that new technologies such as marker-assisted selection are a tool that assists with traditional selection but cannot replace it, as it reduces the time and costs associated with traditional selection.

## 5. Conclusions

This systematic review has critically evaluated SNPs associated with (1) leptin and leptin receptor proteins and their relationship to IMF deposition, and (2) calpain/calpastatin system polymorphisms influencing meat tenderness. Through a comprehensive analysis of these molecular markers’ mechanisms of action, we demonstrate that identifying high-frequency genetic variants associated with economically significant traits (e.g., tenderness, growth rate) can significantly enhance the precision of swine breeding programs. However, our findings highlight three critical research gaps requiring further investigation:

First, expanded genomic studies across diverse porcine breeds are necessary to establish robust genotype–phenotype correlations for marbling and tenderness traits. Second, the polygenic nature of meat quality characteristics demands consideration of epistatic interactions and polygenic scores rather than single-marker analyses. Third, while marker-assisted selection provides valuable tools for genetic improvement, optimal meat quality outcomes require integrated approaches combining advanced genomic technologies, precision nutrition protocols, and welfare-optimized management practices.

The evidence presented underscores that genetic selection constitutes a necessary but insufficient condition for comprehensive meat quality enhancement. Future research directions should prioritize multivariate analyses that account for the complex interplay between genomic factors, production systems, and post-mortem processing parameters.

## Data Availability

The data presented in this study are available in this paper.
